# *Inocellia* (*Amurinocellia*) *calida* (Raphidioptera, Inocelliidae) was first observed as a predator of *Monochamussaltuarius* (Coleoptera, Cerambycidae) in China, the vector of *Bursaphelenchusxylophilus* (Aphelenchida, Aphelenchoididae)

**DOI:** 10.3897/BDJ.12.e114294

**Published:** 2024-01-17

**Authors:** Miao Yu, Jue Wang, Wenfeng Yan, Shiyu Kuang, Yanan Zheng

**Affiliations:** 1 College of Forestry, Shenyang Agricultural University, Shenyang, China College of Forestry, Shenyang Agricultural University Shenyang China

**Keywords:** Inocellia (Amurinocellia) calida, *Monochamussaltuarius*, morphology characters, feeding habit

## Abstract

*Monochamussaltuarius* Gebler (Coleoptera, Cerambycidae) serves as the primary carrier of *Bursaphelenchusxylophilus* (Steiner & Buhrer) (Aphelenchida, Aphelenchoididae) in the middle-temperate zone of China. Pine wilt disease caused by *B.xylophilus* leads to serious losses to pine forestry around the world. It is necessary to study the biological control of *M.saltuarius* to effectively prevent the further spread of *B.xylophilus*. To explore the insect resources that act as natural enemies of *M.saltuarius*, investigations were conducted on natural enemy insects by splitting *Pinuskoraiensis* Siebold & Zucc (Pinales, Pinaceae) damaged by *M.saltuarius* and dissecting their trunks in Yingpan Village, Fushun County, Fushun City, Liaoning Province, China, in 2023. A larva of Inocellia (Amurinocellia) calida (H. Aspöck & U. Aspöck) (Raphidioptera, Inocelliidae) was discovered in the trunk of an infested *P.koraiensis*. Additionally, the feeding habits of *I.calida* were preliminarily examined under indoor conditions and a description of its morphological characteristics was provided. When placed in an indoor environment, the *I.calida* larva began pupating after a period of 21 days, during which time it consumed and attacked a total of 23 *M.saltuarius* larvae. Ultimately, after a pupal period of ten days, the *I.calida* larva emerged successfully as an adult. This discovery marks the first recorded presence of *I.calida* in Liaoning Province and the first documentation of *I.calida* in China, serving as a natural predatory enemy of *M.saltuarius*.

## Introduction

Pine wilt disease is a worldwide forest disease caused by *Bursaphelenchusxylophilus* Steiner & Buhrer (Aphelenchida, Aphelenchoididae) (Mamiya and Kiyohara 1972, Zhao et al. 2008) which has been listed as a quarantine pest in more than 40 countries (Tóth 2011). *Bursaphelenchusxylophilus* is currently distributed in China, Korea, Japan, the United States, Portugal and other countries (Mota and Vieira 2009). *Bursaphelenchusxylophilus* can cause devastating damage to pine forest ecosystems and biodiversity in infected areas and serious economic losses (Ye 2019). Although *B.xylophilus* is seriously harmful, it cannot spread by itself and needs to be transmitted through wounds caused by vector insects feeding and oviposition (Fan et al. 2021). *Monochamussaltuarius* Gebler (Coleoptera, Cerambycidae) is the vector insect of the *B.xylophilus* in Japan (Heisuke et al. 1987), Korea (Kwon et al. 2006) and the mid-temperate region of China (Fan et al. 2021) and carries *B.xylophilus* that spread causing a large number of pine trees to die. The use of natural enemies for biological control is recognised as one of the effective control techniques (Tang et al. 2011) and, as the prerequisite for this is to identify the species of natural enemies, it is necessary to carry out surveys of natural enemies. Several predatory natural enemies of *M.saltuarius* have been documented, including *Stenagostusumbratilis* (Coleoptera, Elateridae), *Trogossitajaponica* (Coleoptera, Trogossitidae), *Thanasimuslewisi* (Coleoptera, Cleridae) and *Kolibaciasquamulata* (Coleoptera, Trogossitidae). In addition to these predators, seven parasitic natural enemies have been identified, namely *Cleonymusserrulatus* (Hymenoptera, Pteromalidae), *Dolichomituscephalotes* (Hymenoptera, Ichneumonidae), *D.curticornis* (Hymenoptera, Ichneumonidae), *D.nakamurai* (Hymenoptera, Ichneumonidae), *Echthrusreluctator* (Hymenoptera, Ichneumonidae), *Dastarcushelophoroides* (Coleoptera, Bothrideridae) and *Rhimphoctonalucida* (Hymenoptera, Ichneumonidae). Furthermore, several unidentified species of the genus *Spathius* (Hymenoptera, Braconidae) are known to parasitise *M.saltuarius*. However, this number remains lower compared to the 27 species of predatory natural enemies and 22 species of parasitic natural enemies recorded for *M.alternatus* (Zhang et al. 2022). Therefore, further exploration and investigations are imperative to discover a more extensive range of natural enemy insect resources.

The species Inocellia (Amurinocellia) calida (H. Aspock & U. Aspock) (Raphidioptera, Inocelliidae) (Aspöck and Aspöck 1973), which was discovered in this study, belongs to the family Inocelliidae within the order Raphidioptera (Engel 2002). This order is characterised by having the fewest number of species amongst fully metamorphosed insects (Oswald and Machado 2018). All extant snakeflies of the Raphidioptera are found only in the Northern Hemisphere (Oswald 2022). Inocelliidae are holometabolous terrestrial and arboreal insects, with carnivorous larvae and adults, making it a rare and valuable species (Oswald 2022, Yang and Liu 2022). Previous research on *I.calida* is limited, with only a few reports indicating its distribution in China (Jilin Province), North Korea, South Korea and the Far East region of Russia (Liu et al. 2009). However, the life history and feeding habits are not reported.

This study is the first to report that *I.calida* is a predator of *M.saltuarius* in China. Additionally, ecological information and specimen photos of *I.calida* were presented.

## Material and Methods

### Insect collection

In March 2023, *P.koraiensis* infected by *B.xylophilus* and its vector *M.saltuarius* were cut into one-metre-long logs in Yingpan Village, Fushun County, Liaoning Province, China (41°56′17″N, 124°12′17″E, 140 m altitude, 823 mm annual average precipitation). These logs were then dissected to investigate and collect the natural enemies of *M.saltuarius*. The number of *M.saltuarius* collected in each log, as well as the natural enemy insects and their predation status, were recorded.

#### Identification and photography

We only found one female of *I.calida*, its examination being carried out using a Zeiss Stemi508 stereomicroscope and the species identification was based on naturally dried specimens. Specimens were photographed using a Nikon D610 camera and Tuli 100 mm F2.8 lens. The species of natural enemy insects were identified using morphological methods (Liu et al. 2009, Shen et al. 2022).

### Study on the feeding habits of Inocellia calida

**Figure 1.**
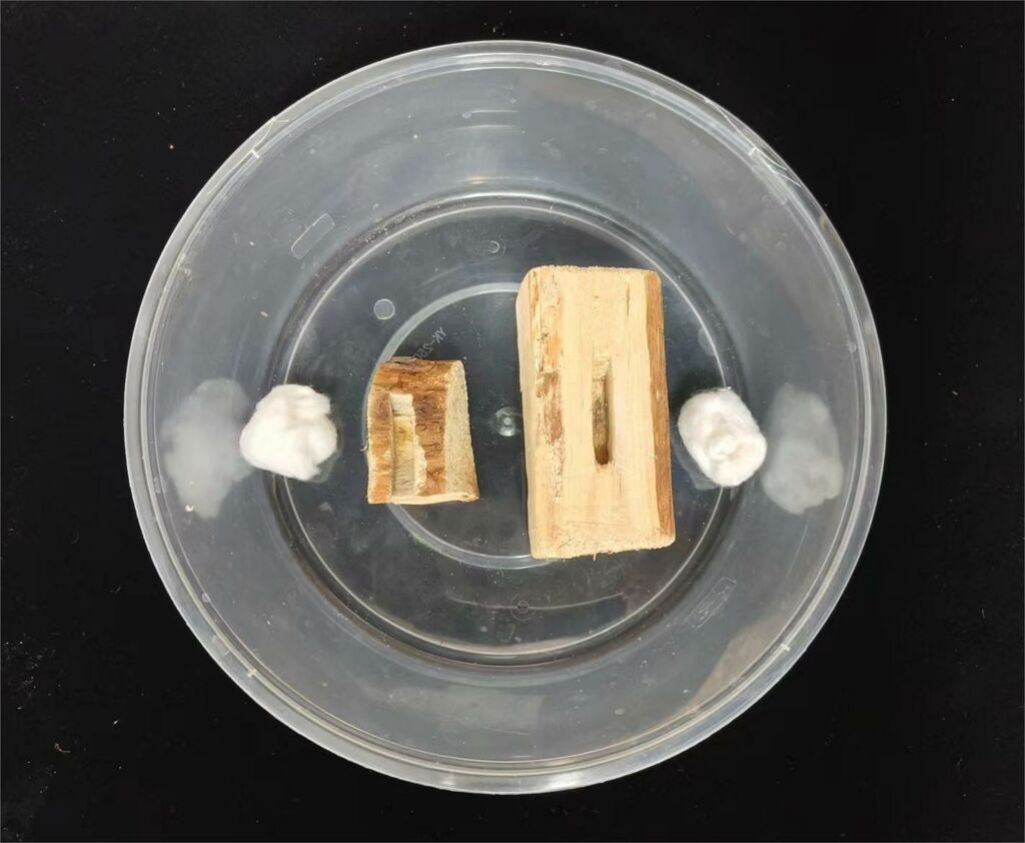
Artificial rearing of Inocellia (Amurinocellia) calida. Including the wood for raising insects, groove for placing *Monochamussaltuarius* larvae, and absorbent cotton.

For the insect-raising experiment, logs of *P.koraiensis* measuring 100 × 50 × 50 mm were used. A middle groove with dimensions of 30 × 10 × 10 mm was carved into one side of each log. Larvae, pupae and adults of *M.saltuarius* were placed in the grooves of the insect-raising logs, while *I.calida* larvae were placed in separate logs. These two sets of logs were then placed inside a transparent plastic box (Zhang et al. 2008). To maintain humidity levels, the box was supplemented with water-soaked absorbent cotton, which was regularly replaced (Fig. 1). During the experiment, one larva of *M.saltuarius* was provided as prey for the *I.calida* larvae each time. After the consumption of the *M.saltuarius* larva, a new one was introduced. The predation behaviour of *I.calida* larvae on *M.saltuarius* larvae was observed daily and the predation ability and the quantity of prey consumed by *I.calida* were recorded.

## Results

### Inocellia (Amurinocellia) calida

Inocellia (Amurinocellia) calida H. Aspöck & U. Aspöck, 1973: 47. Type locality: Russia (Khabarovsk in Amur-Region).

**Figure 2.**
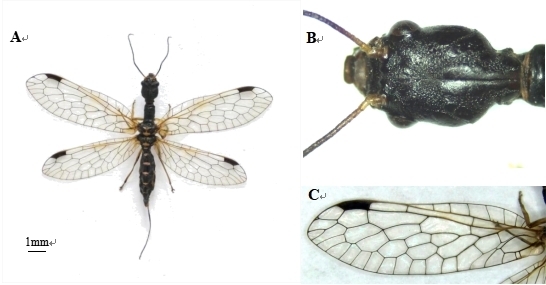
Inocellia (Amurinocellia) calida female. **A** dorsal aspect; **B** head, dorsal aspect; **C** fore wing.

Female: Body length 12.90 mm, body width 2.60 mm, wing length 26.04 mm (Fig. 2).

### Feeding habit

**Figure 3.**
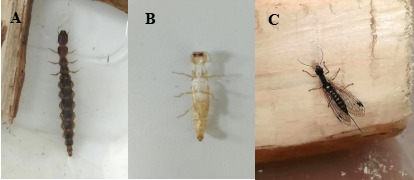
Photographs of Inocellia (Amurinocellia) calida during development. **A** larva; **B** the exuvia of the larva; **C** an adult.

**Figure 4.**
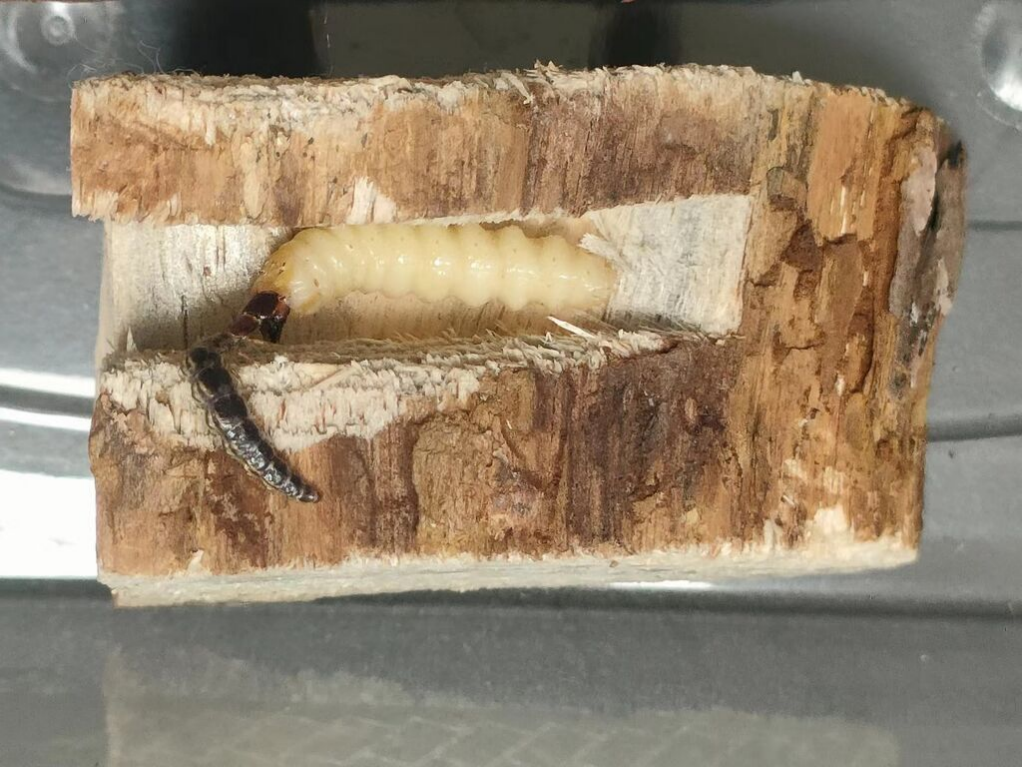
Inocellia (Amurinocellia) calida preying on the larva of *Monochamussaltuarius*.

**Figure 5.**
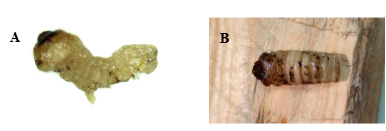
The dead larva of *Monochamussaltuarius*. **A** preyed larva of *Monochamussaltuarius*; **B** the bitten larva of *Monochamussaltuarius*.

The larva of *I.calida* (Fig. 3A) from the Dahuofang Forest presented remarkable activity, by rapidly locating and either preying upon or biting *M.saltuarius* larvae within the worm-hole (Fig. 4). The larva of *I.calida* were collected on 27 March 2023 and, throughout its 21-day artificial feeding larval phase, it predominantly engaged in feeding and excreting during the night-time, capturing or biting a total of 23 *M.saltuarius* larvae. Of these, 16 larvae were preyed upon and seven larvae were bitten. During the indoor rearing phase, the larvae of *I.calida* underwent one moulting process on 16 April 2023 (Fig. 3B). Its pupation period spanned 10 days and turned into an adult on 26 April 2023. Post-emergence (Fig. 3C), it lived for 17 days and died on 16 May 2023. Based on the observations made during the artificial feeding of *I.calida* and the number of larvae it attacked, it became evident that *I.calida* plays a substantial role as a predatory antagonist to *M.saltuarius*. It exhibits a significant predation rate, frequently directly consuming *M.saltuarius* larvae (Fig. 5A) or puncturing their epidermis to extract bodily fluids. In certain cases, a single *M.saltuarius* larva displayed as many as 29 bite marks on its body surface (Fig. 5B).

## Discussion

Previous research has indicated that Inocelliidae are predatory insects with a broad prey range (Aspöck et al. 1991). Inocelliidae larvae typically inhabit bark and primarily feed on various insects, including Coleoptera, Lepidoptera and Hymenoptera, as well as the eggs and larvae of Collembola, mites and spiders (Kovarik et al. 1991, Aspöck and Aspöck 2009). However, there is currently a lack of research on the feeding habits and quantity of Inocelliidae. In this study, over a 21-day period of larva observation, *I.calida* larvae preyed upon and bit 23 *M.saltuarius* larvae. Comparatively, *Cryptalausberus* (Coleoptera, Elateridae), a predator of *M.alternatus*, preyed upon and bit 27 *M.alternatus* larvae throughout its entire 130-day developmental period (Zhang et al. 2008). This indicates that *I.calida* exhibits a higher feeding rate. Given its extended larval development period, *I.calida* has the potential to be an effective biocontrol agent against *M.saltuarius*.

Previous studies have documented that Inocelliidae larvae undergo 10-11 instars (Aspöck et al. 1991). In this study, the *I.calida* larva was fed for 21 days, but as it was already a larva when collected, its exact larval period could not be accurately determined. *I.calida* has a pupal period of 10 days and an adult period of 17 days. Since the larvae in this study moulted once and then began pupation in the pupal chamber within the insect-raising log, it can be inferred that they were in their final instar at the time of collection and have been actively preying before reaching that stage. Most Inocelliidae larvae require a period of static hibernation to complete their life cycle (Kovarik et al. 1991). If the final larval stage is not exposed to a decrease in temperature, it remains alive, active, feeds and moults, but after weeks or months, it moults into a peculiar larva with more or less pronounced pupal characteristics. These larvae may undergo additional moults, but eventually they perish (Aspöck et al. 2018). Interestingly, the *I.calida* individuals reared in this study did not undergo low-temperature stimulation during the larval stage, yet they successfully developed into healthy adults after normal pupation. This phenomenon may be attributed to factors such as abundant food, a safe feeding environment and a small sample size.

In this research, *I.calida* was discovered in *P.koraiensis* logs infected by *B.xylophilus* and *M.saltuarius*. This marks the first documented occurrence of *I.calida* in Liaoning Province and as a predator of *M.saltuarius*. This study was conducted under artificial conditions and the feeding range and amount of *I.calida* larvae under natural conditions were not yet clear. In our experiments, *I.calida* was provided with larvae, pupae and adults of *M.saltuarius*, but it primarily targeted the larvae for biting. Further research is needed to explore the developmental duration, predation targets and feeding preferences of *I.calida* in its natural habitat.
